# Gemini-Assisted Deep Learning Classification Model for Automated Diagnosis of High-Resolution Esophageal Manometry Images

**DOI:** 10.3390/medicina60091493

**Published:** 2024-09-13

**Authors:** Stefan Lucian Popa, Teodora Surdea-Blaga, Dan Lucian Dumitrascu, Andrei Vasile Pop, Abdulrahman Ismaiel, Liliana David, Vlad Dumitru Brata, Daria Claudia Turtoi, Giuseppe Chiarioni, Edoardo Vincenzo Savarino, Imre Zsigmond, Zoltan Czako, Daniel Corneliu Leucuta

**Affiliations:** 1 Second Medical Department, “Iuliu Hatieganu” University of Medicine and Pharmacy, 400006 Cluj-Napoca, Romania; popa.stefan@umfcluj.ro (S.L.P.); ddumitrascu@umfcluj.ro (D.L.D.); andreipopdr@gmail.com (A.V.P.); abdulrahman.ismaiel@yahoo.com (A.I.); lilidavid2007@yahoo.com (L.D.); 2 Faculty of Medicine, “Iuliu Hatieganu” University of Medicine and Pharmacy, 400012 Cluj-Napoca, Romania; brata_vlad@yahoo.com (V.D.B.); turtoidariaclaudia@gmail.com (D.C.T.); 3 Il Cerchio Med Global Healthcare, Verona Center, 37100 Verona, Italy; chiarioni@hotmail.com; 4 UNC Center for Functional GI and Motility Disorders, University of North Carolina at Chapel Hill, Chapel Hill, NC 27599, USA; 5 Gastroenterology Unit, Department of Surgery, Oncology and Gastroenterology, University of Padua, 35128 Padova, Italy; edoardo.savarino@unipd.it; 6 Faculty of Mathematics and Computer Science, Babes-Bolyai University, 400347 Cluj-Napoca, Romania; imre.zsigmond@ubbcluj.ro; 7 Computer Science Department, Technical University of Cluj-Napoca, 400114 Cluj-Napoca, Romania; zoltan.czako@cs.utcluj.ro; 8 Department of Medical Informatics and Biostatistics, “Iuliu Hatieganu” University of Medicine and Pharmacy, 400349 Cluj-Napoca, Romania; danny.ldc@gmail.com

**Keywords:** Gemini, deep learning, esophageal motility disorder diagnosis, image classification, artificial intelligence, HREM

## Abstract

*Background/Objectives:* To develop a deep learning model for esophageal motility disorder diagnosis using high-resolution manometry images with the aid of Gemini. *Methods:* Gemini assisted in developing this model by aiding in code writing, preprocessing, model optimization, and troubleshooting. *Results:* The model demonstrated an overall precision of 0.89 on the testing set, with an accuracy of 0.88, a recall of 0.88, and an F1-score of 0.885. It presented better results for multiple categories, particularly in the panesophageal pressurization category, with precision = 0.99 and recall = 0.99, yielding a balanced F1-score of 0.99. *Conclusions:* This study demonstrates the potential of artificial intelligence, particularly Gemini, in aiding the creation of robust deep learning models for medical image analysis, solving not just simple binary classification problems but more complex, multi-class image classification tasks.

## 1. Introduction

Esophageal motility disorders (EMDs), characterized by abnormal contractions of the esophageal body (hypertensive, hypotensive, failed, or spastic) or impaired relaxation of the lower esophageal sphincter, can lead to a spectrum of symptoms, including dysphagia, chest pain, and regurgitation [1]. Among the diagnostic modalities employed to assess esophageal motility, high-resolution esophageal manometry (HREM) has emerged as a gold standard, providing detailed information about pressure changes along the length of the esophagus during swallowing [1,2,3,4].

Despite the clinical utility of HREM, the interpretation of the studies remains challenging and strongly related to the observer’s experience in manometry testing. Indeed, manual analysis of esophageal manometry images involves identifying specific patterns of the peristaltic waves and classifying them according to established criteria. This process demands expertise, and it is time-consuming and highly susceptible to inter-observer variability [5,6,7,8]. Furthermore, the increasing prevalence of EMD and the growing demand for effective healthcare require innovative solutions to streamline a rapid automated diagnostic process. Consequently, there is a compelling need for automated solutions that can enhance the efficiency, consistency, and accuracy of EMD diagnosis.

Artificial intelligence (AI) has revolutionized various fields of medicine, demonstrating the potential to enhance diagnostic accuracy and efficiency. In recent years, the application of AI in medical imaging has gathered significant attention, showcasing promising results in tasks such as image segmentation, classification, and disordered pattern recognition. In the realm of EMD, there exists a compelling opportunity to harness the power of AI for the automated diagnosis of HREM images.

Gemini, unveiled in December 2023 by Google DeepMind, specifically the Gemini 1.5 Flash model, constitutes a novel family of multimodal large language models, encompassing variants like Gemini Ultra, Pro, and Nano. Positioned as a successor to LaMDA and PaLM 2, it leverages its multimodal capabilities to reason seamlessly across various data modalities, including image, video, audio, and code. This versatility, coupled with its proficiency in natural language processing, empowers Gemini to act as a multifaceted tool, facilitating tasks ranging from information retrieval and reasoning to creative text generation and code comprehension [9,10,11,12].

While not currently designed for direct code generation, Gemini’s capabilities position it as a valuable tool in the development of deep learning (DL) programs for automated image recognition and diagnosis in medicine. Its ability to access and process vast amounts of information allows it to effectively research existing medical knowledge, relevant datasets, and established DL architectures. This knowledge can then fuel the exploration of specific medical imaging tasks and potential solutions. For instance, a user could query Gemini about common chest X-ray findings associated with pneumonia, and Gemini could identify relevant datasets and research papers detailing convolutional neural network (CNN) architectures effective in pneumonia detection [12,13]. Furthermore, Gemini’s ability to understand and generate different creative text formats, like code comments or pseudocode, could be harnessed to document the thought process and decision-making behind building the DL model. This would not only enhance collaboration and knowledge sharing among developers but also improve the transparency and interpretability of the model, crucial aspects for trust and acceptance in the medical field.

Additionally, as the field of DL in medicine evolves, Gemini can continuously learn and update its knowledge base, staying abreast of the latest advancements and contributing to the development of increasingly accurate and reliable automated diagnosis tools, potentially leading to earlier interventions and improved patient outcomes. It is important to note, however, that Gemini’s role is not to replace human expertise but rather to augment it by providing efficient information retrieval, exploration, and documentation capabilities, ultimately accelerating the development and responsible application of DL solutions in medical image analysis.

In the context of healthcare, the most important advantage of Gemini compared to other similar models (like ChatGPT-4o) is its capability to integrate and analyze diverse data types. Combining images with text enhances the understanding of model decisions for clinical trust. Gemini is being increasingly integrated into medical research, particularly in the domain of automatic diagnosis from medical images, leveraging advanced DL systems. This application involves training neural networks on large datasets of annotated medical images, to recognize patterns indicative of various pathologies [14,15,16]. By utilizing these models, Gemini assists researchers in interpreting complex visual data, providing preliminary diagnoses, and suggesting potential areas of concern. The system’s ability to process and analyze vast amounts of image data rapidly enhances diagnostic accuracy and efficiency, reducing the cognitive load on clinicians [14,15,16]. Furthermore, its integration into diagnostic workflows supports real-time decision-making, enabling more personalized and timely patient care. Ongoing research focuses on refining these DL models to improve their sensitivity, specificity, and generalizability across diverse patient populations, while addressing challenges related to data privacy, model interpretability, and the potential for bias [8,9,10]. The continued development of Gemini-based diagnostic tools holds promise for advancing the field of medical imaging and improving patient outcomes through more precise and automated diagnostic processes [14,15,16].

This study addresses these challenges by introducing a novel Gemini-assisted Deep Learning Classification Model (G-DLCM) for automated HREM image diagnosis. The G-DLCM leverages the unique capabilities of the Gemini large language model (LLM) in several ways: (a) data augmentation: Gemini helped us to improve the training data by suggesting enhancements and supplying the sample code, resulting in a more comprehensive and diverse dataset for better generalization and robustness; (b) feature engineering: the LLM utilizes its extensive expertise in medical terminology and esophageal physiology to select the most suitable ML models for extracting useful features from HREM data. It suggests the usage of CNN models which have inbuilt feature engineering capabilities; the DL model then uses these features to improve pattern recognition and categorization accuracy; (c) explainable AI (XAI): Gemini assisted our team in selecting the most appropriate algorithm for interpreting image classification results generated by DL models. Based on the prompts and the problem requirements, Gemini selected the LIME algorithm and provided code samples that can be used to translate complex DL model outputs into insights interpretable by clinicians, fostering trust and acceptance in the G-DLCM. For this reason, the aim of this study was to assess the feasibility of a Gemini-assisted DL model for automated HREM image classification. We evaluated the model’s accuracy, sensitivity, and specificity in disease diagnosis, aiming to establish its potential for improving diagnostic efficiency and accuracy.

## 2. Materials and Methods

### 2.1. Data Collection

The database of HREM from our manometry department was used for this study and the images were collected between October 2014 and February 2021. The database contained records from symptomatic patients, referred to manometry for esophageal symptoms like dysphagia, chest pain, heartburn, or regurgitation. The protocol of examination, the algorithm, and the classification of EMDs, was based on Chicago v3.0 recommendations, which were in use at the time. The protocol included an EGJ baseline recording of 2 min, followed by ten 5 mL wet swallows, spaced at more than 30 s. Manometry was performed early in the morning, after at least 6 h of fasting, in the supine position with the thorax angulated at 30°. The manometry system used was commercialized by ISOLAB (Standard Instruments GmbH, Karlsruhe, Germany) and used a solid-state catheter with 36 sensors (Unisensor^®^, Zurich, Switzerland).

**Figure 1:**
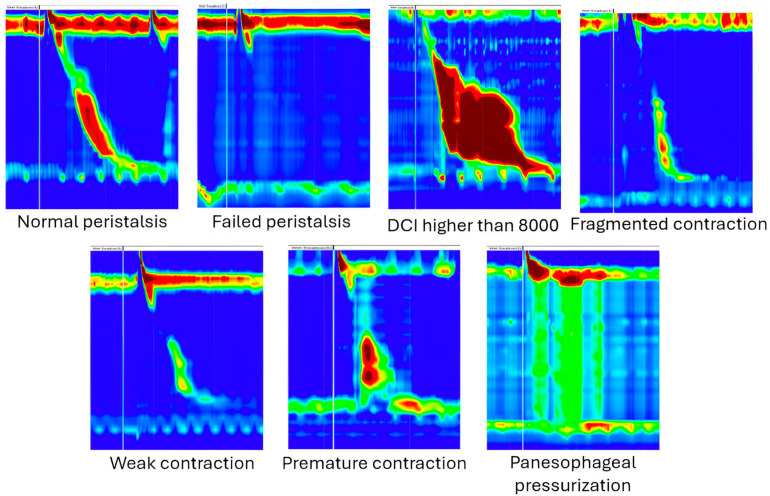
Examples of swallowing pattern classes. In high resolution manometry images blue colour represents areas with very low pressure (arround 0 mmHg, such as observed in the thorax). The upper and the lower esophageal sphincter, as well as the contraction of the esophageal body after a swallow determine higher pressure zones and are coded in yellow, red or dark red, depending on the value of the recored pressure.

The images were selected and labeled by two specialists (T.S.B. and S.L.P.) from the same department. In cases of disagreement, the images were labeled in collaboration with two analog specialists from another department (G.C., E.V.S.). All images were classified based on Chicago version 3.0 criteria. The software enables the storing of photos representing 60 s of the recording. The test swallows were marked by a white vertical line (placed during the recording), and the swallow that followed just after this white line was labeled by the human experts. All the swallows contained no other markers (i.e., for esophageal sphincters) and were therefore raw swallows. The dataset contained a total of 926 images belonging to seven classes (see examples in Figure 1). Each test swallow was classified and labeled in one of the following categories, irrespective of IRP value, based on the aspect (which refers to contraction vigor, contraction patterns, or intrabolus pattern) observed in the esophageal body:

- Normal peristalsis—swallows without large breaks (>5 cm), with a normal distal latency (≥4.5 s) and a normal contraction vigor, as measured by a distal contractile integral (DCI) between 450 and 8000 mmHg·s·cm;
- DCI higher than 8000 for all swallows, with DCI > 8000 mmHg·s·cm; therefore, this is classified by human experts as hypercontractile swallows;
- Fragmented contractions—contractions with DCI > 450 mmHg·s·cm, but with large break (>5 cm length) in the 20 mmHg isobaric contour;
- Weak contractions—contractions with DCI > 100 mmHg·s·cm but <450 mmHg·s·cm;
- Premature contractions—contractions with DL < 4.5 s and DCI > 450 mmHg·s·cm;
- Panesophageal pressurization—for swallows followed by a uniform pressurization of >30 mmHg extending from the upper to the lower esophageal sphincter;
- Failed peristalsis—contractions with DCI < 100 mmHg·s·cm.

Our department is a referral center for achalasia patients; hence, we had many images classified as failed peristalsis or panesophageal pressurization. Unfortunately, the classes were not well balanced, containing 139 images of normal peristalsis, 21 of DCI higher than 8000, 58 of fragmented contractions, 54 of weak contractions, 27 of premature contractions, 390 of panesophageal pressurization, and 237 images of failed peristalsis. To solve this issue, we used traditional data augmentation techniques, like random cropping, scaling, etc.

### 2.2. Gemini Assistance

Gemini offered advice on preprocessing procedures, including scaling, normalizing pixel values, and using data augmentation techniques. It aided in selecting the best network design, algorithm, and model optimization, recommending appropriate hyperparameters and troubleshooting code-related problems to ensure efficient development. It also helped us explain the results of the trained model by suggesting the most appropriate algorithms and enabling us to apply the selected algorithm to the results of the CNN model. Classifying swallowing disorders is one of the most important steps in diagnosing EMDs, but to obtain the final class, we also need the classification of the integrated relaxation pressure, and as the last step, we must combine these results by applying the Chicago classification algorithm.

### 2.3. Experimental Setup

We used the TensorFlow [17] library to implement and train a machine learning model. The model’s training was conducted on a local computer with 32 GB of RAM, an Intel i710750H CPU running at 2.60 GHz with 6 cores and 12 logical processors and a GPU of type Nvidia Tesla K80 with 12 GB VRAM, manufactured by HP in Palo Alto, CA, USA. We adhered to Gemini’s suggestions for developing the model, including the essential libraries and packages for our DL model.

### 2.4. Gemini-Aided Creation of a DL Model for HREM Image Classification

First, we asked Gemini to explain the steps for classifying medical images belonging to multiple classes. The given steps were the following:

(1) Data acquisition and preprocessing—Acquire a comprehensive library of high-resolution manometry photos from diverse sources such as hospitals and research databases to guarantee broad applicability. Implement domain-specific preprocessing techniques such as eliminating background noise and standardizing image dimensions to enhance model performance.
(2) Feature extraction—Utilize convolutional neural networks (CNNs) to extract distinctive features from unprocessed photos automatically. To enhance performance and decrease training duration, use pre-trained CNN models such as VGGNet [18] or ResNet [19], which are fine-tuned for your task.
(3) Model selection and training—Choose a multi-class classification technique appropriate for the size of your dataset, the types of features present, and the performance metrics you aim to achieve. Partition your dataset into training (70%), validation (20%), and test (10%) subsets to avoid overfitting and guarantee impartial evaluation.
(4) Evaluate and refine—Assess the model’s performance using relevant metrics like accuracy, precision, recall, F1-score, and confusion matrix.
(5) Deployment and impact—Create a user-friendly interface for clinicians to use the model and understand its predictions. Perform prospective studies to verify the model’s effectiveness in actual clinical environments.

In the following steps, we asked Gemini to give us example implementations for each step enumerated above. Based on the swallowing patterns, the available images were manually moved to separate folders. As Gemini suggested, we also split the whole dataset into three subsets, so we created a training folder containing approximately 70% of the images and two other folders for validation (20%) and testing (10%). We imported the images using the Image Data Generator class from *tensorflow.keras.preprocessing.image*. The images were resized to 1/255, which converted the pixel values to the [0, 1] interval. This preprocessing step is crucial because most currently available CNN models work with values between 0 and 1. In the model creation stage, we used transfer learning. Based on the problem description, Gemini suggested the usage of EfficientNet [20] trained on the ImageNet [21] dataset by retraining its fully connected layers for our problem. We added our own fully connected layer consisting of one layer with 256 neurons and one with 7 for the seven different swallowing pattern classes we want to classify: aperistalsis, hypertensive contractions (DCI over 8000), fragmented, weak, panesophageal pressurization, premature contractions, and normal peristalsis. Example images for each of these classes can be seen in Figure 1.

**Figure 2:**
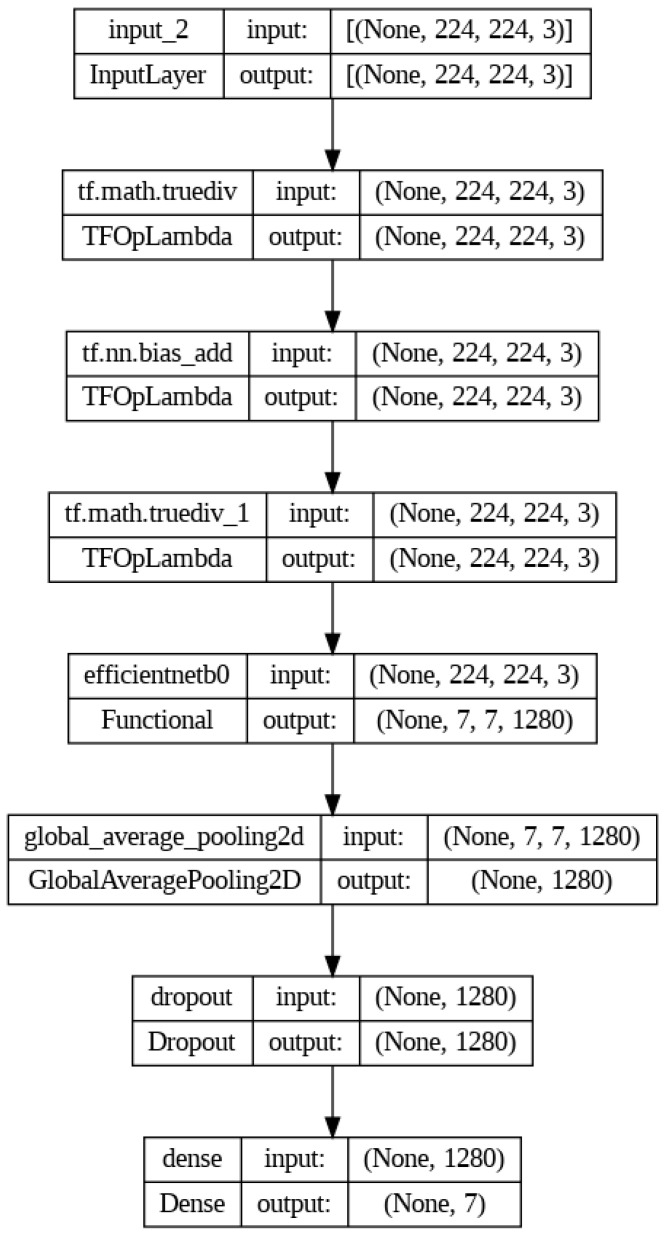
The architecture of the CNN model used for HREM image classification.

A dropout rate of 0.5 was added to ensure the model did not overfit. Moreover, the model was finetuned by gradually unfreezing a subset of the neural layers of the base model and retraining it multiple times. The architecture of the created CNN model is seen in Figure 2. In the training phase, we utilized the Adam [22] optimizer, a widely used and efficient technique for DL applications. The model was compiled using the Adam optimizer, categorical cross-entropy loss function, and categorical accuracy metric. We trained the model for 250 epochs with no finetuning, then five times for another 20 epochs using finetuning, and then we gradually retrained 20 extra layers in each step.

## 3. Results

### 3.1. Model Performance

**Figure 3:**
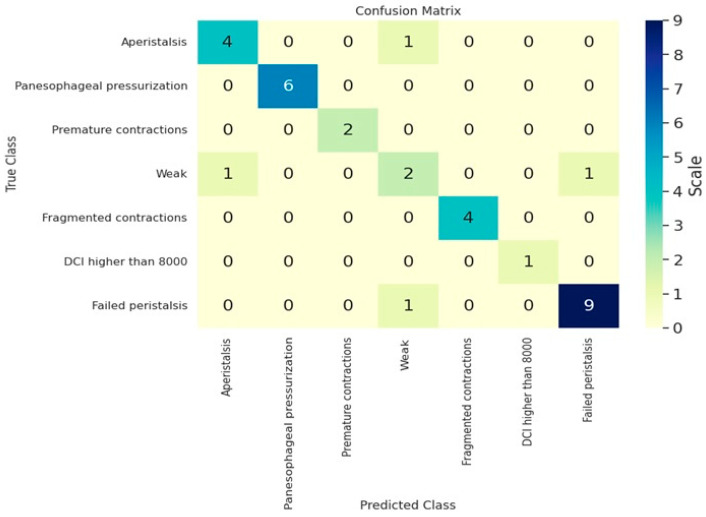
Confusion matrix for esophageal body contraction patterns.

The model’s performance demonstrates noteworthy outcomes across different metrics. The model’s accuracy, a key metric of its overall quality, is 0.88. Our model accurately predicted 88% of the test data instances, demonstrating high reliability. Table 1 presents the average performance metrics for the testing set. We assessed the model’s performance post-training using accuracy, precision, recall, and F1-score metrics. Figure 3 displays a confusion matrix, providing a detailed analysis of the model’s performance for each studied swallowing disorder class. Table 2 shows a detailed overview of the performance metrics, showing the results separately for each class. As we can see in this table, the best performances were obtained for the panesophageal pressurization, premature contractions and fragmented contractions classes with an F1-score of 100%, while the worst classification metrics were obtained in the case of the weak class. This class overlaps with most of the other classes, which makes the classification task very hard. In the last step, we experimented with multiple algorithms to explain the results generated by the trained model. The selected algorithm for CNN result explainability will be described in the following section.

### 3.2. Interpreting the Outcomes

**Figure 4:**
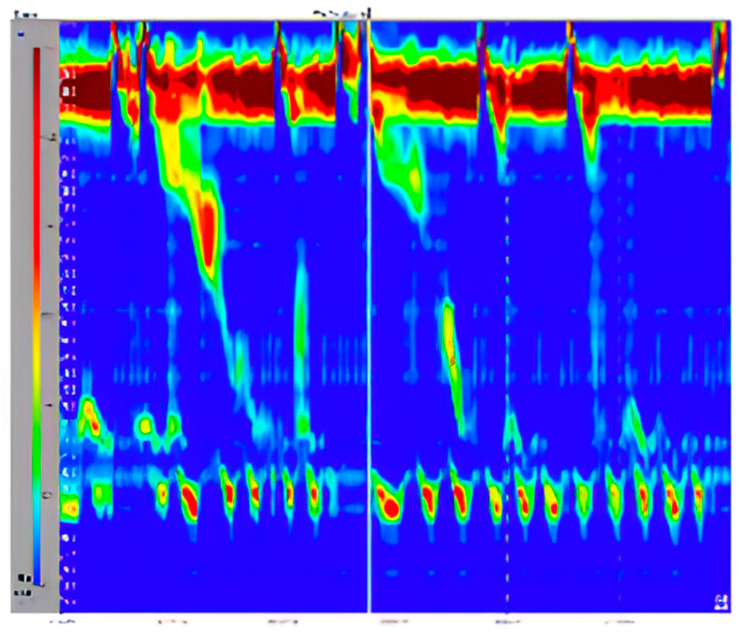
Original image to classify.

**Figure 5:**
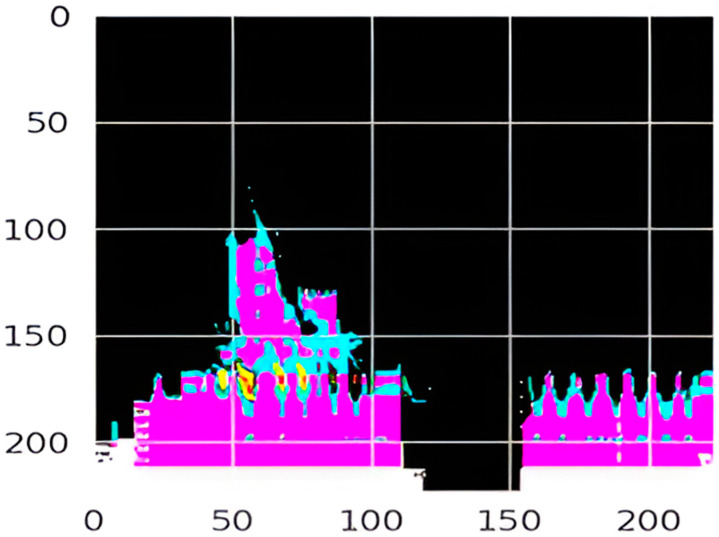
Mask generated by LIME. Different colors show different levels of feature importance.

LIME [23] heatmaps were used to display the characteristics and patterns learned by the CNN. Heatmaps validate the algorithm’s accurate identification of the swallow event’s location in the image for feature extraction, classification, and decision-making (Figure 4 and Figure 5).

**Figure 6:**
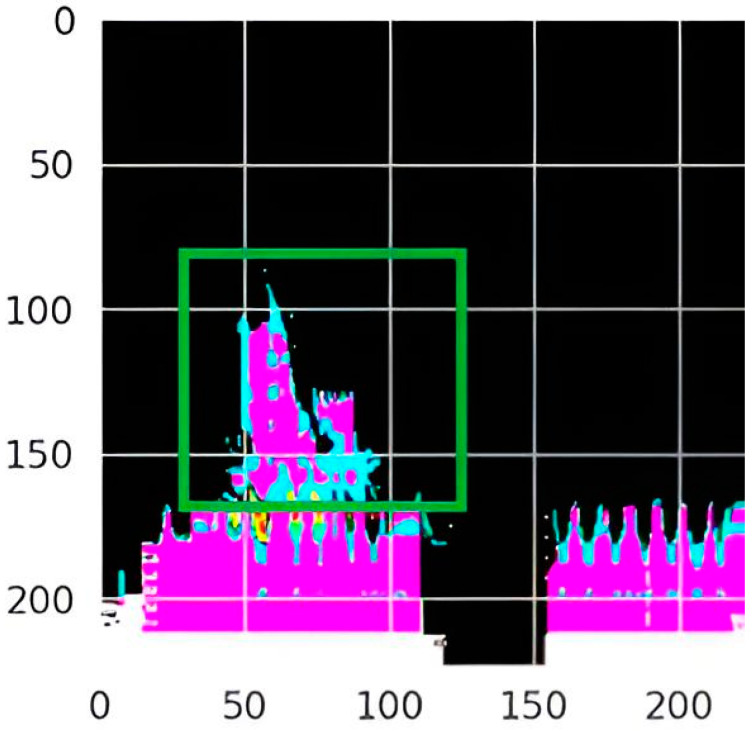
The exact moment of swallowing found automatically by the trained model, marked by the green square.

As shown in the above mask, the model focused on the lower part of the image, which is important because it contains multiple important features and parameters (e.g., the integrated relaxation pressure). In addition, the mask focused on the region highlighted in green in Figure 6, which contains precise timing of swallowing. This demonstrates that the model is focusing on the right regions from the images when classifying the swallowing patterns.

The LIME algorithm aims to understand the model by altering the input data and analyzing the impact of these modifications on the predictions. The concept involves partitioning the image into super-pixels, which are clusters of adjacent pixels that share similar color and brightness. This strategy is plausible because the classification of an image is likely influenced by numerous pixels; therefore, altering a single pixel would have little effect on the forecast. Next, we constructed a collection of perturbed photos by replacing certain image super-pixels with gray super-pixels. We obtained a probability for each of these altered photos, indicating the presence of the target class according to the model. We further trained a basic linear model that is weighted to prioritize perturbed images that closely resemble the original image. Finally, we displayed the super-pixels with the highest weights on the original image to provide an explanation and generate the image mask. In simple words, a LIME heatmap is like a highlighter for the model’s decision. It shows which parts of the data were the most important in making the prediction. Brighter colors mean the model paid more attention to that area when it made its prediction.

## 4. Discussion

Our work aimed to create a strong and precise DL model with the help of Gemini to analyze patterns recorded by HREM in different EMD. Furthermore, we aimed to improve the diagnostic accuracy of HREM for esophageal motility disorders by reducing differences in diagnoses among practitioners with varying levels of training and experience [5]. The use of AI in the case of unclear swallowing images has been reported to enhance diagnostic precision, assist in decision-making, decrease unnecessary invasive treatments, and support personalized therapeutic approaches.

This study’s innovation was fully using the Gemini language model to develop our DL model. We chose Gemini because of its multimodal capabilities, because of which we could use images, and not just a textual explanation of the problem, as input; this way, it gave us more precise and problem-specific answers and not just general guidance. We used the multimodal capabilities for guidance, but the final solution was achieved by unimodal DL models, more specifically by convolutional neural networks, which are specialized in image classification; hence, the results were more precise than using a general model in a specific context.

By leveraging Gemini’s features, we optimized the development process, achieving a high accuracy of 88%. This indicates that Gemini was instrumental in enhancing the model. Additionally, using Gemini promoted transparency and reproducibility, potentially reducing doctors’ and nurses’ workload and improving diagnostic accuracy. The study is unique and robust due to its novel integration of traditional medical imaging methods with cutting-edge AI. This is the first time Gemini has been used in this way, significantly improving EMD. Gemini also assisted us in making key decisions, such as selecting the algorithm. The analysis provided insights into the optimal network structure for our research and detailed different choices with thorough explanations. Using different evaluation metrics, including accuracy, precision, recall, F1-score, and confusion matrix, provides a comprehensive evaluation of the model’s performance. Using LIME heatmaps to reveal the model’s decision-making process demonstrates a dedication to transparency and interpretability, which is crucial for building trust among clinicians and patients. The integration streamlined the model creation process and improved efficiency.

We utilized a CNN as the DL framework for our research, based on guidance from Gemini. CNNs necessitate notably less preprocessing in comparison to other classification techniques. Unlike conventional approaches, CNNs may autonomously learn filters or features without manually constructing filters. We used regularization techniques like dropout and weight decay to tackle overfitting. Early stopping is a regularization method used in deep neural networks to halt training when parameter updates cease to provide improvements on a validation set. The strategy involves storing and updating the best parameters during training. Training finishes when parameter updates no longer increase performance after a specified number of iterations and the last best values are used. This functions as a regularizer by confining the optimization process to a reduced parameter space. Gemini provided tremendous assistance by offering suggestions in this way. By optimizing functions such as dropout, weight decay, and L2 regularization, the generalization potential of our model was dramatically enhanced. Comprehending the decision-making process of DL models is essential in healthcare settings to establish trust and promote using AI-assisted models. Techniques like Grad LIME [23], SHAP [24], or CAM [25] can be used to improve the interpretability and explainability of a model, ensuring that its predictions are consistent with human expert knowledge.

We utilized LIME heatmaps, as recommended by Gemini, to gain significant insights into our model’s decision-making process. LIME provides a graphical representation of complex DL models. This strategy enables a more profound comprehension of models during detection or prediction tasks. We confirmed the model’s attention to pertinent features by visualizing the informative sections of the photos. This method helps to enhance the interpretability and explainability of DL models.

Gemini not only helped with the technical aspects of developing DL models but also played a crucial role in resolving code-related issues during our study. The challenges included resolving syntax mistakes, addressing problems related to data processing and data formatting, and implementing multiple deep learning libraries and frameworks. Gemini played a crucial role in fixing issues related to incorporating TensorFlow and Keras [26] into our DL model. We used it to detect and rectify flaws in our model’s structure, use optimization techniques, and configure hyperparameters. Gemini assisted us in determining the most efficient methods for data augmentation, normalization, and encoding. We asked Gemini about the best way to augment our dataset and it provided multiple sample code snippets that we could use to create new images based on the existing dataset. The data augmentation techniques that we used are random cropping of the edges and scaling. It also suggested to use conditional GANs or variational autoencoders to generate new realistic data, but in our case, it was enough to only apply traditional data augmentation techniques. Trying these more advanced data augmentation DL models can be a good candidate for future work to increase the precision of the trained CNN.

One of the limitations of Gemini is that it sometimes generates code that is not directly executable, containing logical bugs or syntactic errors. Furthermore, it can use nonexistent methods from different libraries. Another issue is that it does not consider the library version, so it generates code that uses functions from diverse and incompatible versions. Because of these concerns, a highly experienced specialist must review and adjust the code to make it runnable. At this level, Gemini represents a valuable coding assistant. It can reduce the time needed to develop a new algorithm or train a new model but cannot resolve a problem end-to-end without human coordination. Another concern is that we have limited transparency of Gemini’s training data, which means we cannot know if the generated code is an exact copy or a modified replica of code written by someone else, which limits its usage in commercial products. By incorporating a multidisciplinary strategy with domain experts and machine learning professionals, the development process may become rigorous and free from biases. Using Gemini, we can reduce the development cost by reducing the number of specialists working on a problem or a project. Still, we cannot eliminate human input, so domain specialists and programmers are still relevant in innovative work.

In our previous studies, we developed an AI system to automate HREM diagnosis. The system recognized correct probe positioning with high accuracy, as well as discriminating IRP levels and identifying esophageal peristalsis patterns using the Chicago algorithm [27,28,29].

A review by Fass et al. showed that AI can automate the interpretation of image-based esophageal tests, particularly high-resolution manometry (HRM) and functional luminal imaging probe (FLIP) studies, with high accuracy in identifying landmarks, determining HRM adequacy, and distinguishing achalasia from non-achalasia patterns [27], but AI applications are less advanced in ambulatory reflux monitoring due to data assimilation challenges from multiple impedance and pH channels [27].

The integration of AI into medical imaging has witnessed transformative strides, demonstrating significant capabilities in disease detection, characterization, and prognostication. Notably, the application of AI in radiology, pathology, and cardiology has set a precedent for its potential in enhancing diagnostic accuracy and expediting patient care. In the context of esophageal motility disorders, the fusion of AI and HREM holds promise in changing the diagnostic landscape [28,29,30].

Machine learning algorithms, particularly those based on deep neural networks, excel at learning intricate patterns from large datasets. By training these algorithms on annotated HREM images, it becomes feasible to automate the recognition and classification of nuanced pressure patterns indicative of esophageal motility disorders. The ability of AI to process information swiftly and consistently offers a compelling solution to the challenges associated with manual HREM analysis.

While the concept of automated diagnosis in esophageal manometry is still in its infancy, pioneering research endeavors have laid the groundwork for its potential implementation. Studies exploring the feasibility of AI in HREM interpretation have demonstrated promising results in the identification of abnormal pressure patterns associated with various esophageal motility disorders.

However, challenges persist, including the need for large and diverse datasets for robust algorithm training, validation, and testing. Additionally, the generalizability of AI models across different patient populations and the incorporation of real-time diagnostic capabilities into clinical workflows remain areas warranting further investigation.

DL techniques offer promising potential in automating HREM image diagnosis by analyzing HREM tracings [31,32,33]. While existing DL models show promise, challenges remain: limited annotated HREM data hinder generalizability, the inherent complexity of HREM data poses challenges for model development and interpretation, and the lack of explainability in DL models limits clinical adoption.

The successful development and validation of the G-DLCM can have significant implications for gastroenterology practice. They can be summarized as follows: (a) improved diagnostic accuracy and efficiency: automated EMD diagnosis using the G-DLCM can provide faster and more objective results, potentially reducing diagnostic errors and delays; (b) enhanced accessibility and affordability: the model can be deployed in resource-constrained settings, improving diagnostic capabilities, and reducing healthcare costs; also, the development costs can be reduced by using Gemini as a coding assistant; (c) support for clinical decision-making: by providing accurate and interpretable predictions, the G-DLCM can empower clinicians to make informed decisions regarding treatment options and patient management.

CNNs, like those used in this work, are powerful tools, but their effectiveness is heavily reliant on the quality and consistency of the data they are trained on. Problems can arise when human labelers have different understandings of categories, leading to inconsistent labels for the same data point or human errors in image labeling can mislead the model. Also, imbalanced data can have a negative impact on the per class precision. During the training, the model could focus only on the classes with an abundance of images and neglect the classes with few examples. This is why choosing the right metrics is also critical when working with machine learning models. For example, the models have a lower precision in the case of the weak class, which can be explained by the small number of exiting images used for training and by inter-class similarity.

Developing a machine learning model to classify esophageal motility disorder images is a significant step towards improving diagnosis accuracy and efficiency. However, to realize the full potential of the model, it is crucial to integrate it into an end-to-end clinical workflow. This integration ensures seamless interaction between the model and healthcare professionals, leading to improved patient care. But this integration requires high effort, because the algorithm must comply with multiple regulatory standards. Data should be handled securely, the algorithm requires explainability, and it also involves high of costs, like developing a user-friendly and intuitive interface. Additionally, the training of healthcare professionals, continuous improvement, and feedback must be integrated, etc.

## 5. Conclusions

Our research emphasizes the capability of AI-supported DL models, such as those created using Gemini, in medical image interpretation.

In this work, we used Gemini as a coding assistant, and it gave us valuable answers and guidance on solving the problem of classifying HREM images. Using the code samples provided by Gemini, we were able to build and train a highly accurate model for classification and we used the LIME algorithm to explain the results generated by the trained CNN model.

Using Gemini to create HREM image classification DL model is a novel approach, and the results obtained demonstrate the efficiency and the usability of this LLM in healthcare and in the development of new DL/ML models.

The obtained classification model may assist doctors and motility labs in their everyday work, minimize the variability between observers, and save money and time on repeated duties. The usage of Gemini has the potential of reducing development costs by decreasing the number of hours needed by a programmer to build and train novel DL/ML models.

## Figures and Tables

**Table 1:** Classification report for swallowing disorders.

Metric	Result
Accuracy	0.88
Precision	0.89
Recall	0.88
F1-score	0.885

**Table 2:** Classification metrics for each swallowing disorder class.

	Precision	Recall	F1-Score
0	0.80	0.80	0.80
1	1.00	1.00	1.00
2	1.00	1.00	1.00
3	0.50	0.50	0.50
4	1.00	1.00	1.00
5	1.00	1.00	1.00
6	0.90	0.90	0.90
accuracy			0.88
macro avg	0.89	0.89	0.89
weighted avg	0.88	0.88	0.88

## Data Availability

Data are available on request due to restrictions, e.g., privacy or ethical. The data are not publicly available due to the sensitive nature of medical data.
